# Cadaveric Analysis of Key Anatomic Structures of Athletic Pubalgia

**DOI:** 10.5435/JAAOSGlobal-D-23-00070

**Published:** 2023-06-14

**Authors:** Ryan O'Donnell, Steven DeFroda, Steven L. Bokshan, James G. Levins, Michael J. Hulstyn, Ramin R. Tabaddor

**Affiliations:** From the Department of Orthopaedic Surgery, Brown University, Warren Alpert School of Medicine, Providence, RI.

## Abstract

This study proposes to establish in-depth inspection of the anatomic structures involved with the pathology of athletic pubalgia in a cadaver model.

**Methods:**

Eight male fresh frozen cadavers were dissected in a layered fashion. The rectus abdominis (RA) and adductor longus (AL) tendon insertions were isolated to quantify the size of the anatomic footprint and distance from the surrounding anatomy.

**Results:**

The RA insertional footprint was 1.65 cm (SD, 0.18) in width by 1.02 cm (SD, 0.26) in length, and the AL insertional footprint on the underside of the pubis was 1.95 cm (SD, 0.28) in length by 1.23 cm (SD, 0.33) in width. The ilioinguinal nerve was 2.49 cm (SD, 0.36) lateral to the center of the RA footprint and 2.01 cm (SD, 0.37) lateral to the center of the AL footprint. The spermatic cord and the genitofemoral nerve were just lateral to the ilioinguinal nerve and were 2.76 cm (SD, 0.44) and 2.66 cm (SD, 0.46) from the rectus and AL footprints, respectively.

**Conclusion:**

Surgeons should be cognizant of these anatomic relations during both initial dissection and tendon repair to optimize repair and avoid iatrogenic injury to critical structures in the anterior pelvis.

Athletic pubalgia or “sports hernia” can be a serious core injury for athletes. Athletic pubalgia usually involves a tear of the rectus abdominis (RA) or adductor longus (AL) muscle group from the pubis.^1^ However, there is a lack of clarity in the literature regarding the exact injury patterns, surgical indications, surgical techniques, and location of relevant surgical anatomy for anatomic footprint of the structures involved in sports hernia. The anatomy of the anterior pelvis and groin is complex, which may account for the confusion surrounding the pathoanatomy and treatment of athletic pubalgia.^2,3^ Historically, athletic pubalgia has been classified and confused with many other clinical entities including “sportsman hernia,” “Gilmore groin,” osteitis pubis, “hockey groin syndrome,” “Ashby's inguinal ligament enthesopathy,” and others.^2,4,5^

While many complex anatomic regions of sports medicine such as the posterolateral knee and capsular ligaments of the shoulder have been further classified by anatomic studies, few cadaveric anatomical studies have examined the pubis and the muscular attachments thought to play a role in athletic pubalgia.^6,7,8,9,10,11,12,13,14^ Previous literature reported the adductor attachment anatomy the adductor/rectus anatomic relationship, and “triangular pyramidalis muscle” on the anterior abdominal wall attached to the linea alba bilaterally, albeit with less attention to the surgical implications of these relationships.^15-17^ To date, there has been another previous study to characterize the structures of the anterior pelvis involved in athletic pubalgia in a more comprehensive manner.^18^ Riff et al determined the footprint of the RA, inguinal ligament, AL, adductor brevis, pectineus, gracilis, and overall aponeurotic plate. Other key structures they investigated were the genital branch of the genitofemoral nerve, obturator nerve, and femoral artery and vein.^18^ This study examines the exact anatomic position and footprint anatomy of the structures involved in sports hernia (RA and AL), particularly with reference to the pubis and adjacent neurovascular anatomy. We also look to investigate the exact location of the ilioinguinal nerve, spermatic cord, and superficial inguinal ring relative to the RA and AL footprints because these structures are in close proximity, may be implicated in the pathoanatomy of athletic pubalgia, and were not investigated in the previous study by Riff et al.^18,19,20,21,22^ We hypothesized that while major neurovascular structures are relatively safe when considering the surgical approach for the management of athletic pubalgia, vital neurovascular anatomy is within the surgical field, closer than some surgeons may be aware.

## Methods

### Dissection

Funding for cadaveric specimens was obtained from the Rhode Island Hospital Orthopaedic Foundation. Dissections were done in 8 fresh frozen male cadavers by three of the authors (R.O., S.D., R.T.), one of whom is a fellowship-trained sports medicine specialist (R.T.). Only male cadavers were studied as to keep the anatomy consistent between all specimens. Each specimen included tissue from 1 cm above the umbilicus to the level of the mid-femur on bilateral lower extremities. The mean specimen age was 67 years (range 55 to 83 years). Cadaveric specimens were thawed overnight before dissection. Dissection was begun with a midline incision and bilateral transverse incisions across the inguinal folds. The skin and subcutaneous fat were excised from the level of anterior superior iliac spines to the mid-thigh, exposing all superficial fascia including the anterior rectus sheath, external oblique fascia, and fascia lata. Dissection was continued in a layer-by-layer fashion starting midline at the pubic symphysis and working laterally, bilaterally. The RA and AL were dissected first. We did note a large aponeurosis connecting the two tendinous insertions, which has been previously described.^16^ The pyramidalis muscle was found overlying the recut sheath as previously described.^16^ Dissection was done laterally, isolating the inguinal ligament attaching to the pubic tubercle, the spermatic cord exiting the superficial inguinal ring, and the ilioinguinal nerve exiting on the medial side of the spermatic cord through the superficial inguinal ring. The genitofemoral nerve was identified behind and deep to the spermatic cord. Moving laterally, the femoral neurovascular bundle and the obturator nerve were carefully dissected out and identified. The attachment sites of the AL and RA were sharply removed from the pubis with a scalpel, and the corresponding footprints were measured. Careful attention was placed to try to identify the tendonous insertion footprint only and not the entire cartilaginous plate. The center point of the AL and RA attachments were identified by measuring the central point of the cross-sectional area of the footprint. Each specimen dissection was done bilaterally.

### Statistical Analysis

**Figure 1:**
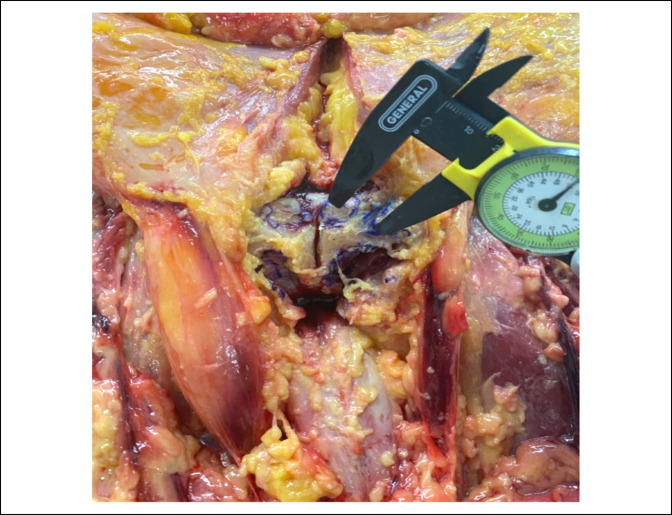
Image showing an example of measurement with a caliper from the central point of the rectus abdominus tendon insertion and the pubic tubercle.

All measurements were made using a 0.01-mm dial caliper (Grainger) (Figure 1). The caliper was zeroed in between each measurement. Mean distance values and SDs across specimens were calculated for each measurement using Microsoft Excel 2020 (Microsoft).

## Results

### Dissection

**Figure 2:**
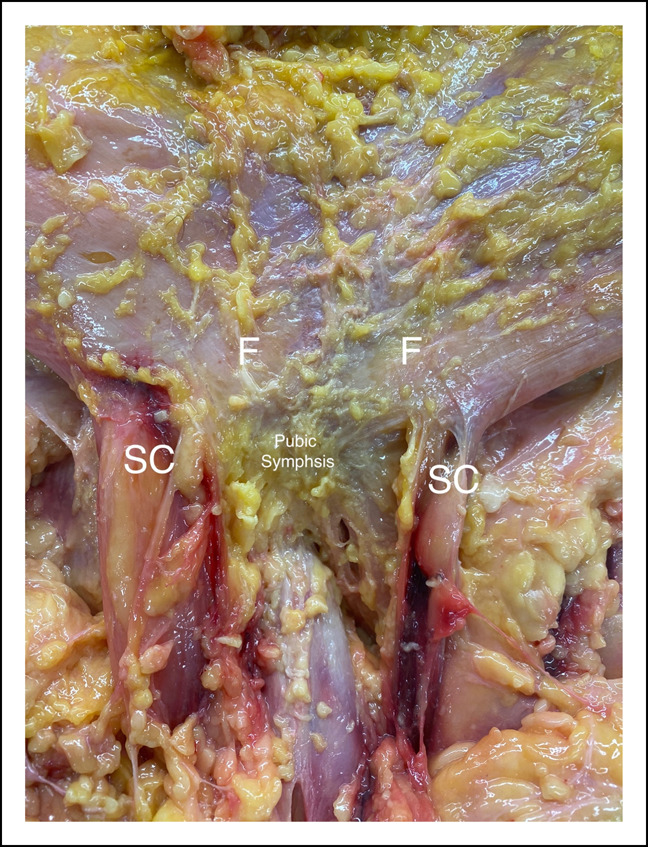
Photograph depicting the fascial tissue over the external oblique and rectus fascia after overlying skin and subcutaneous fat have been removed. F = fascial tissue overlying the external oblique and rectus abdominis, SC = spermatic cord

The confluence of the fascia overlying the external oblique and RA was oriented in an oblique direction. This fascia continued to the linea alba (Figure 2). A robust aponeurosis was then noted on all specimens, and this fascia was followed distally toward the pubis and medially to the midline. This aponeurotic layer was used to trace back to the RA and AL tendon attachments. Under the fascia of this aponeurotic plane, a small section of muscle, previously described as the pyramidalis, was noted between the fascia and the underlying RA tendon in all specimens.^16^

### Rectus Abdominis and Adductor Longus

**Figure 3:**
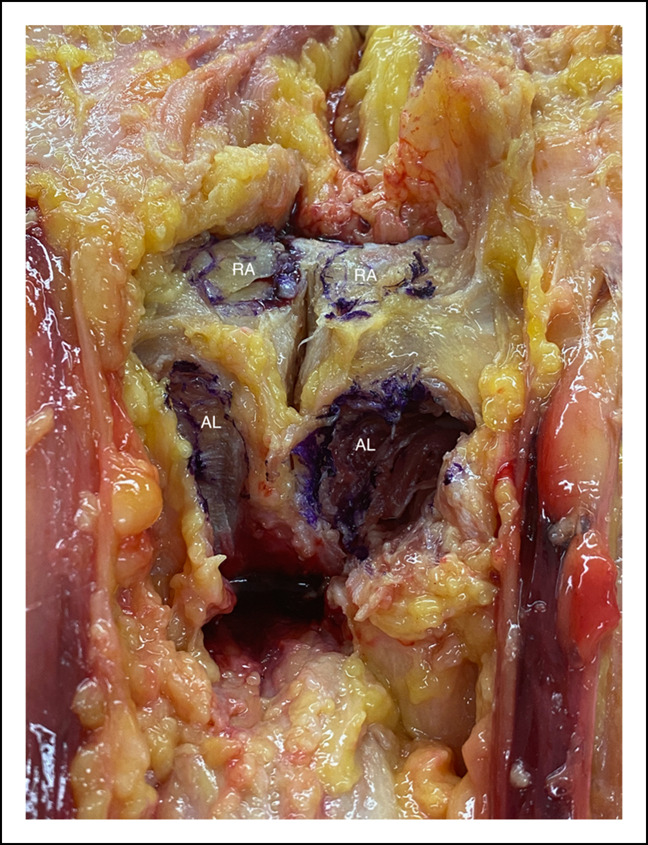
Photograph showing the footprint/attachment sites of the rectus abdominis and adductor longus tendons. AL = adductor longus, RA = rectus abdominis

Measurements of the critical surrounding structures from these muscular attachments are presented in Table 1. The RA insertional footprint measured 1.65 cm (SD, 0.18) in width by 1.02 cm (SD, 0.26) in length, on average. The footprint was found to be mostly oval shaped; however, three cadavers had a more circular appearance (similar length and width) (Figure 3). The AL insertional footprint on the underside of the pubis was 1.95 cm (SD, 0.28) in length by 1.23 cm (SD, 0.33) in width. This footprint was more semilunar in shape as shown in Figure 3. The central point of the RA footprint was located 0.78 cm (SD, 0.30) from the pubic symphysis. The central point of the AL footprint was located 1.03 cm (SD, 0.36) from the pubic symphysis.

**Table 1:** Measurement of Anatomic Landmarks From the Rectus Abdominis (RA) and Adductor Longus (AL) Footprints

Structure	Average (cm)	SD
Rectus abdominis footprint size (length)	1.02	0.18
Rectus abdominis footprint size (width)	1.65	0.26
Rectus abdominis footprint to pubic symphysis	0.78	0.30
Adductor longus insertion size (width)	1.23	0.33
Adductor longus insertion size (length)	1.95	0.28
Adductor longus footprint to pubic symphysis	1.03	0.36
Ilioinguinal ligament attachment/pubic tubercle to rectus footprint	1.50	0.27
Ilioinguinal ligament attachment/pubic tubercle to adductor longus footprint	2.06	0.42
Spermatic cord to RA footprint	2.76	0.44
Spermatic cord to AL footprint	2.66	0.46
Superficial inguinal ring to RA footprint	4.23	0.63
Superficial inguinal ring to AL footprint	5.37	0.67
Ilioinguinal nerve to RA footprint	2.01	0.37
Ilioinguinal nerve to AL footprint	2.49	0.36
Femoral NV bundle to AL footprint	6.32	1.01
Femoral NV bundle to RA footprint	6.41	0.81

### Critical Structures

**Figure 4:**
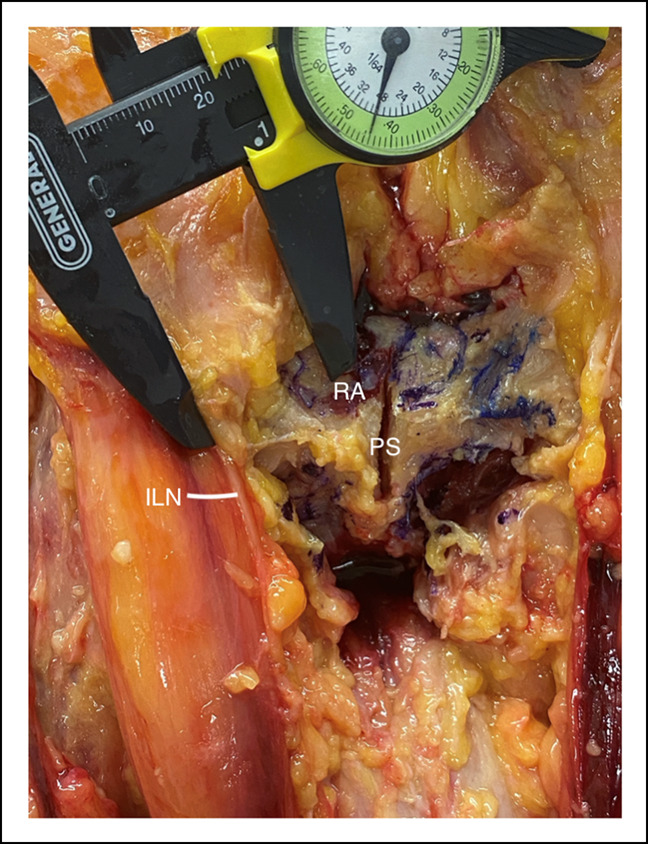
Photograph showing the measurement from the central point of the rectus abdominis footprint to the ilioinguinal nerve. ILN = ilioinguinal nerve, PS = pubic symphysis, RA = rectus abdominis

**Figure 5:**
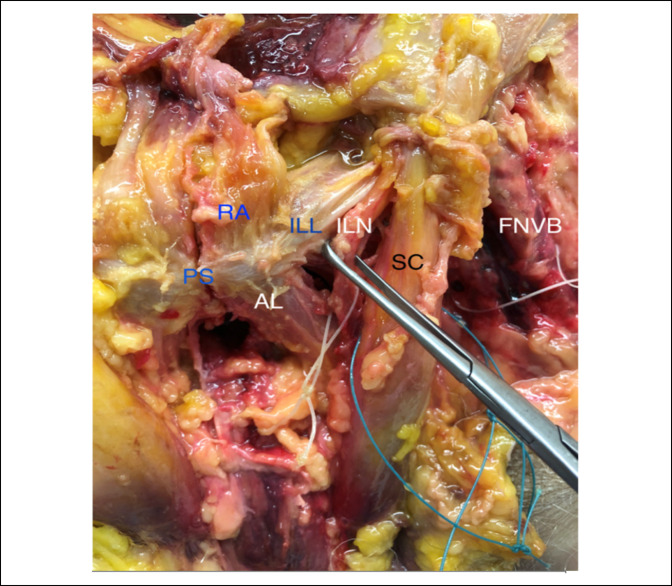
Photograph showing the anterior pubis with all critical structures surrounding the pathologic structures in athletic pubalgia before take down of the rectus abdominis and adductor longus tendons. AL = adductor longus, FNVB = femoral neurovascular bundle, ILL = ilioinguinal ligament, ILN = ilioinguinal nerve, PS = pubic symphysis, RA = rectus abdominis, SC = spermatic cord

**Figure 6:**
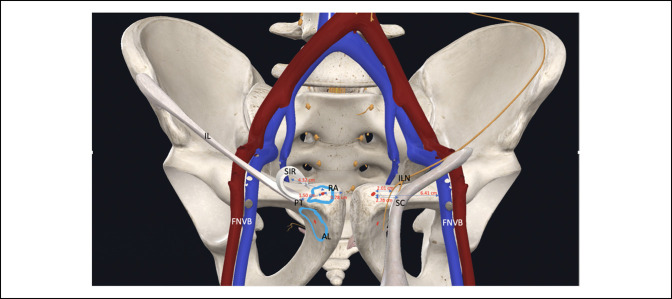
Illustration demonstrating the footprints of the rectus abdominis and adductor longus with nearby critical structures. Measurements to the RA footprint included. Key: blue outline = footprints of RA (superior) and AL (inferior), red dot = central point of the footprint, IL = inguinal ligament, ILN = ilioinguinal nerve, FNVB = femoral neurovascular bundle, PT = pubic tubercle, SC = spermatic cord, SIR = superficial inguinal ring

The spermatic cord was found 2.76 cm (SD, 0.44) laterally from the center of the RA footprint and 2.01 cm (SD, 0.37) lateral to the center of the AL footprint. The spermatic cord was found exiting the superficial inguinal ring, which was 4.23 cm (SD, 0.63) and 5.37 cm (SD, 0.67) laterally from the RA and AL footprints, respectively. The ipsilateral pubic tubercle (ilioinguinal ligament attachment site) was 1.50 cm (SD, 0.27) and 2.06 cm (SD, 0.42) lateral from the RA and AL footprints, respectively. The ilioinguinal nerve was reliably found exiting the superficial inguinal ring medial to the spermatic cord and was 2.49 cm (SD, 0.36) lateral to the center of the RA footprint and 2.01 cm (SD, 0.37) lateral to the center of the AL footprint (Figure 4). This nerve was found to have multiple, smaller cutaneous branches exiting distally and superficially which were reliably encountered during the approach and layered dissection. At the superficial inguinal ring, the ilioinguinal nerve was medially to the spermatic cord. This relationship continued until it passed the AL footprint, where the structures crossed and the spermatic cord traveled more medially as it descended toward the testicle. The genital branch of the genitofemoral nerve was reliably found within the internal spermatic fascia lying deep to and underneath the spermatic cord. Moving more laterally away from the midline, the femoral neurovascular bundle (at its most medial aspect) was 6.41 cm (SD, 0.81) and 6.32 cm (SD, 1.01) lateral to the RA and AL footprints, respectively. The anterior pubis with the critical structures dissected and labeled is shown in both photograph and illustration form in Figure 5 and 6.

## Discussion

Understanding the complex anatomy of the athletic pubalgia footprint is crucial for understanding the pathology, establishing safe landmarks, and developing the knowledge of danger zones for both the approach and repair of sports hernias. This study investigates and highlights the proximity of critical structures to both the RA and AL, two of the major pathologic sites involved in athletic pubalgia. The RA insertional footprint measured 1.65 cm (SD, 0.18) in width by 1.02 cm (SD, 0.26) in length, and its central point was 0.78 cm (SD, 0.30) from the pubic symphysis. The AL insertional footprint on the underside of the pubis was 1.95 cm (SD, 0.28) in length by 1.23 cm (SD, 0.33) in width, and its central point was 1.03 cm (SD, 0.36) from the pubic symphysis. Riff et al^18^ found the average length and width of the RA footprint to be 5.3 and 2.1 cm, respectively, which is considerably smaller than this study's results. This difference can likely be explained because we attempted to measure only the tendon footprint and not the entire aponeurotic plate. The size of the AL footprint was very similar to previous studies.^15,18^ Given that orthopaedic surgeons may enlist the services of a general surgeon for the surgical approach for this technique, this study offers vital anatomic information for those who treat this pathology.

Historically, athletes with long-standing groin pain were thought to have “true” hernias which were causing their symptoms. In the early 1990s, studies described an insufficiency of the posterior inguinal wall consistent with a direct inguinal hernia in soccer and rugby players.^23,24^ However, these studies were retrospective in nature. More recently, there is an increasing body of literature to suggest that the critical area of symptomatic pathology is the AL and RA insertional anatomy. Adductor tendon injuries are the most common cause of groin pain in athletes, accounting for up to 62% of cases.^25,26^ Accordingly, a popular treatment option for athletic pubalgia has been AL tenotomy.^26,27,28,29,30^ The RA also seems to play a large role in the pathology of athletic pubalgia as well.^2,31,32^ In a large prospective study with nearly 4 years of follow-up data, Meyers et al^29^ had a 95% success rate using an open approach to reattach the inferolateral edge of the RA to the pubic bone in what they termed “pelvic floor repair.” The external oblique aponeurosis is also thought to play a role in pathogenesis if injured and has been implicated in hockey groin syndrome.^19,31^

In addition to various surgical procedures, several imaging studies have used both MRI and ultrasonography to attempt to identify injuries and relevant anatomy of the groin in athletes with varying results.^33-35^ Zoga et al^33^ found a pattern of a high incidence of both RA and adductor tendon injury on MRI in patients seeing a subspecialist for groin pain. In another series, attenuation of the abdominal wall musculofascial layers and increased signal in groin muscles were found in 27 of 30 patients and had a 95% correlation with surgical findings.^36^

Taylor et al found success with operating on athletes with presumed RA tears at the site of the pubis in 1991.^31,32^ As they describe it, surgical intervention for these patients consisted of pelvic floor repairs, which was a “broad surgical reattachment of the inferolateral edge of the RA muscle with its fascial investment to the pubis and adjacent anterior ligaments.”^1,31^ In a series of 276 high-performance athletes, one study found that 88% of those with lower abdominal pain had concomitant adductor pain. AL tenotomy, either through a mini-open technique or with concomitant sports hernia repair, has been an effective treatment with excellent return-to-sport results in recreational and profession athletes.^21,28^ In our study, we demonstrated the exact size of the footprint of these tendons and the distance from the pubic symphysis.

During initial dissection, we noted the fascial layer of the external oblique, which became more robust in nature as it came midline and distal toward the pubis. The external oblique fascial layer is involved in the pathogenesis of groin pain in hockey players. Irshad et al^19^ found all patients in a series of 22 National Hockey League players to have tearing of the external oblique aponeurosis. The RA-AL aponeurotic plate has been described by Emblom et al, where MR imaging showed increased signal in the aponeurosis and commonly a partial tendon avulsion underneath the tissue on the inferior edge of the superior pubic rami. A “adductor-to-rectus flap” with repair of the aponeurosis was done in their series of 100 athletes, with improved patient-reported outcomes and return to play.^37^ Underneath this aponeurotic plate, we did find a small muscular layer on top of the RA tendon, which has been described as the pyramidalis muscle.^16,30^

A complete understanding of the location of surrounding neurovascular structures is essential for diagnostic purposes and preventing surgical complications. The ilioinguinal nerve was the closest neurovascular structure to the RA and AL footprints, 2.01 and 2.49 cm, respectively. The ilioinguinal nerve causes chronic groin pain in patients with previous open-mesh inguinal hernia repair by either entrapment or trauma. There is debate whether to divide or preserve this nerve during inguinal hernia repairs.^38,39^ The ilioinguinal nerve can be an important part of the pathoanatomy in athletic pubalgia. Irshad et al^19^ found branches of the ilioinguinal nerve emerging from tears in the external oblique aponeurosis in all professional hockey players with chronic groin pain. Ilioinguinal nerve compression has been found in athletic pubalgia, and radiofrequency denervation of the nerve and ilioinguinal ligament can improve symptoms, at least in the short term.^20^ Along with the ilioinguinal nerve, the nearby genitofemoral nerve, which we identified within the internal spermatic fascia lying deep to the spermatic cord, is a cause of neuropathic pain and is many times attributed to iatrogenic injury during previous hernia repair.^40^ Riff et al^18^ found the genital branch of the genitofemoral nerve 4.3 ± 1.5 cm from the inguinal ligament footprint. In this study, the genital branch of the genitofemoral nerve was reliably found deep to the spermatic cord, which was 2.76 (SD, 0.44) cm and 2.66 (SD, 0.46) cm from the RA and AL footprints, respectively. Currently, there is no consensus of how to best treat the ilioinguinal nerve at the time of sports hernia repair. Some cohort studies have conducted nerve ablation or neurotomy^19,41^ while others have not specifically addressed it.^1,4,31^ Regardless of the treatment technique, it is critical to know the exact distances of these structures from the repair site.

This study is not without limitations. This was a cadaveric study of uninjured patients, and as such, the distances between structures may vary in injured, pathologic tissue compared with healthy tissue. However, the footprints for the tendons should not change, except for the variation from specimen to specimen. The overall age of our cadavers in this study was more advanced (67 years) than the typical age seen in younger athletes undergoing athletic hernia repair. Only male cadavers were studied as to keep the anatomy consistent between all specimens. Because female patients account for only 5% to 15% of patients with athletic pubalgia,^1,42^ we chose to focus our examination on male specimens only. This eliminated any between-sex differences that may have been observed. Finally, we were unable to isolate and describe the iliohypogastric nerve because this was not included within our cadaveric tissue because of processing of the cadavers. This nerve has been implicated in lower abdominal and pelvic neurogenic pain.^19,22^

## Conclusion

Critical structures such as the ilioinguinal nerve, spermatic cord, and genital branch of the genitofemoral nerve were found in close proximity to the footprints of the RA and AL tendon attachments. Surgeons should be cognizant of the precise distances during both initial dissection and tendon repair to optimize repair as well as avoid iatrogenic injury during athletic pubalgia repair.
